# Supplementary material for: Machine learning derived retinal pigment score from ophthalmic imaging shows ethnicity is not biology
Source: Nat Commun. 2025 Jan 2;16:60. doi: 10.1038/s41467-024-55198-7 (PMC11696055; doi:10.1038/s41467-024-55198-7)
Supplement: Supplementary file 1 — Supplementary Information [file 41467_2024_55198_MOESM1_ESM.pdf]

Supplemental Information:
Machine learning derived retinal pigment score from ophthalmic imaging shows ethnicity is not biology

Supplementary Table 1: Number of fundus photographs ran through the RPS pipeline by dataset.

Dataset	Total	Included	Ungradable
**Overall**	157938 (100.0%)	88896 (56.3%)	69042 (43.7%)
UK Biobank	135592 (100.0%)	74851 (55.2%)	60741 (44.8%)
Epic-Norfolk	16042 (100.0%)	10089 (62.9%)	5953 (37.1%)
ODIR (Chinese)	3098 (100.0%)	208891 (67.45%)	101007 (32.65%)
Tanzanian	2076 (100.0%)	1150 (55.4%)	926 (44.6%)
Australian	1130 (100.0%)	715 (63.3%)	415 (36.7%)

N (row %)

Supplementary Table 2. Baseline UK Biobank patient-level characteristics by tertiles of retinal pigment score (RPS).

		RPS quintiles				
**Characteristic**	**Overall**, N = 44,320	**1**, N = 8,864	**2**, N = 8,864	**3**, N = 8,864	**4**, N = 8,864	**5**, N = 8,864
**Retinal pigment score***	-0.82 (-9.89, 10.39)	-15.00 (-17.28, -13.22)	-8.20 (-9.89, -6.52)	-0.82 (-2.79, 1.19)	7.90 (5.53, 10.39)	19.94 (16.25, 24.96)
**Age**	56 (49, 63)	55 (48, 62)	58 (50, 63)	58 (50, 63)	58 (50, 63)	54 (47, 61)
**Sex**						
Female	24,414 (55%)	4,850 (55%)	4,882 (55%)	4,893 (55%)	4,972 (56%)	4,817 (54%)
Male	19,906 (45%)	4,014 (45%)	3,982 (45%)	3,971 (45%)	3,892 (44%)	4,047 (46%)
**Ethnicity****						
White	40,704 (92%)	8,804 (100%)	8,804 (100%)	8,745 (99%)	8,498 (96%)	5,853 (67%)
Black	1,135 (2.6%)	1 (<0.1%)	2 (<0.1%)	5 (<0.1%)	43 (0.5%)	1,084 (12%)
Asian	1,078 (2.4%)	2 (<0.1%)	4 (<0.1%)	17 (0.2%)	115 (1.3%)	940 (11%)
Mixed	369 (0.8%)	5 (<0.1%)	10 (0.1%)	23 (0.3%)	67 (0.8%)	264 (3.0%)
Chinese	161 (0.4%)	0 (0%)	0 (0%)	1 (<0.1%)	8 (<0.1%)	152 (1.7%)
Other	599 (1.4%)	12 (0.1%)	15 (0.2%)	33 (0.4%)	88 (1.0%)	451 (5.2%)
**Skin color****						
Very fair	3,583 (8.2%)	1,082 (12%)	884 (10%)	745 (8.5%)	550 (6.3%)	322 (3.7%)
Fair	28,889 (66%)	6,692 (76%)	6,489 (74%)	6,229 (71%)	5,743 (66%)	3,736 (43%)
Light olive	8,219 (19%)	936 (11%)	1,295 (15%)	1,631 (19%)	2,123 (24%)	2,234 (26%)
Dark olive	779 (1.8%)	58 (0.7%)	91 (1.0%)	123 (1.4%)	161 (1.8%)	346 (4.0%)
Brown	1,705 (3.9%)	15 (0.2%)	16 (0.2%)	37 (0.4%)	135 (1.5%)	1,502 (17%)
Black	482 (1.1%)	0 (0%)	0 (0%)	1 (<0.1%)	10 (0.1%)	471 (5.5%)
**Hair color****						
Blonde	4,567 (10%)	1,469 (17%)	1,202 (14%)	901 (10%)	685 (7.8%)	310 (3.5%)
Red	1,781 (4.0%)	437 (4.9%)	400 (4.5%)	397 (4.5%)	332 (3.8%)	215 (2.4%)
Light brown	16,657 (38%)	3,985 (45%)	3,875 (44%)	3,671 (42%)	3,226 (37%)	1,900 (22%)
Dark brown	16,656 (38%)	2,621 (30%)	2,977 (34%)	3,350 (38%)	3,953 (45%)	3,755 (43%)
Black	3,980 (9.0%)	214 (2.4%)	273 (3.1%)	405 (4.6%)	556 (6.3%)	2,532 (29%)
Other	486 (1.1%)	110 (1.2%)	112 (1.3%)	104 (1.2%)	84 (1.0%)	76 (0.9%)
**Height (in cm)**	168 (162, 176)	169 (163, 176)	169 (162, 176)	168 (162, 176)	168 (162, 175)	168 (161, 175)
**Townsend index of deprivation (quintiles)****						
1 (least deprived)	13,047 (29%)	2,799 (32%)	2,875 (33%)	2,725 (31%)	2,634 (30%)	2,014 (23%)
2	9,029 (20%)	1,934 (22%)	1,812 (20%)	1,844 (21%)	1,858 (21%)	1,581 (18%)
3	8,447 (19%)	1,655 (19%)	1,715 (19%)	1,705 (19%)	1,715 (19%)	1,657 (19%)
4	8,303 (19%)	1,626 (18%)	1,550 (18%)	1,618 (18%)	1,630 (18%)	1,879 (21%)
5 (more deprived)	5,429 (12%)	838 (9.5%)	894 (10%)	962 (11%)	1,017 (11%)	1,718 (19%)

Median (IQR) for continuous variables.

Count (column %) for categorical variables.

*Patient-level retinal pigment score calculated as the mean score from right and left eyes.

**Variables with missing data: ethnicity (n=274, 0.6%), skin color (n=663, 1.5%), hair color (n=193, 0.4%), Townsend index of deprivation (n=65, 0.1%)

Supplementary Table 3: Standardised difference in Retinal Pigment Score (RPS) per specified differences in covariates for the UK Biobank cohort. P-values are two-sided and calculated with the z-statistic. Estimates are mutually adjusted for all covariates shown in the table. Bold p-values mean statistically significant results. Mean (SD) RPS = 0.94 (13.20).

Characteristic	Beta	95% CI	p-value
**Age (per 5 years)**	0.02	0.01, 0.02	**1.3e-08**
**Sex**			
Female	—	—	

Male	0.01	-0.01, 0.04	0.279
**Ethnicity**			
White	—	—	
Black	1.15	1.07, 1.23	**1.6e-158**
Asian	1.09	1.03, 1.16	**4.0e-216**
Mixed	0.91	0.82, 1.00	**4.7e-86**
Chinese	1.49	1.35, 1.62	**4.6e-103**
Other	0.96	0.88, 1.04	**2.8e-135**
Missing	0.44	0.32, 0.56	**1.4e-13**
**Skin colour**			
Very fair	—	—	
Fair	0.17	0.14, 0.20	**5.9e-27**
Light olive	0.47	0.44, 0.51	**8.2e-147**
Dark olive	0.55	0.48, 0.62	**4.3e-55**
Brown	0.72	0.65, 0.79	**4.3e-88**
Black	0.83	0.72, 0.94	**5.7e-48**
Missing	0.47	0.39, 0.55	**5.2e-32**
**Hair colour**			
Blonde	—	—	
Red	0.26	0.22, 0.31	**4.1e-28**
Light brown	0.16	0.13, 0.19	**5.1e-29**
Dark brown	0.37	0.34, 0.40	**1.2e-136**
Black	0.53	0.49, 0.58	**4.8e-122**
Other	0.17	0.09, 0.25	**1.9e-05**
Missing	0.27	0.13, 0.42	**2.0e-04**
**Refractive status**			
Myopia <-6.00D	—	—	
Myopia	0.12	0.08, 0.17	**1.3e-08**
Emmetropia	0.16	0.11, 0.20	**1.1e-12**
Hyperopia	0.11	0.06, 0.15	**1.1e-06**
Hyperopia >+6.00D	-0.10	-0.22, 0.03	0.133
**Height (per 5cm)**	-0.02	-0.02, -0.01	**3.6e-08**
**Townsend index of deprivation (quintiles)**			
1 (least deprived)	—	—	
2	0.01	-0.01, 0.03	0.480
3	0.01	-0.01, 0.03	0.353
4	0.02	-0.01, 0.04	0.169
5 (more deprived)	0.06	0.03, 0.09	**2.5e-05**
Missing	-0.02	-0.23, 0.19	0.833

Supplementary Table 4: Variance inflation factor testing on final linear regression model.

**Variable**	**Df**	**aGSIF***
Age	1	1.08
Sex	1	1.47
Ethnicity	6	1.16
Refractive status	4	1.01
Height	1	1.47
Townsend quintiles	5	1.02
UKB centre	5	1.01
Skin colour	6	1.15
Hair colour	6	1.09

Df, degrees of freedom; aGSIF, adjusted generalised standard error inflation factor.

*Values greater than 1.6 are considered to introduce strong collinearity.

Supplementary Table 5: Standardised difference in Retinal Pigment Score (RPS) per specified differences in covariates for the UK Biobank cohort stratified by three main ethnic groups from linear regression. P-values are two-sided and calculated with the z-statistic. Estimates are mutually adjusted for all covariates shown in the table. Bold p-values mean statistically significant results.

	White ethnic group[*]			Black ethnic group[*]			Asian ethnic group[*]		
**Characteristic**	**Beta**	**95% CI**	**p-value**	**Beta**	**95% CI**	**p-value**	**Beta**	**95% CI**	**p-value**
**Age (per 5 years)**	0.29	0.22, 0.37	**2.2e-15**	-0.69	-0.98, -0.40	**3.0e-06**	-0.80	-1.10, -0.50	**2.7e-07**
**Height (per 5cm)**	-0.21	-0.30, -0.12	**4.6e-06**	0.07	-0.25, 0.38	0.679	-0.60	-0.99, -0.21	**0.003**
**Sex**									
Female	—	—		—	—		—	—	
Male	0.22	-0.11, 0.54	0.198	-0.91	-2.03, 0.21	0.110	-0.41	-1.88, 1.07	0.588
**Skin colour**									
Very fair	—	—		—	—		—	—	
Fair	2.18	1.76, 2.60	**1.3e-24**	6.10	-1.75, 13.96	0.128	-0.23	-5.16, 4.71	0.928
Light olive	6.33	5.84, 6.82	**1.4e-141**	1.66	-6.51, 9.83	0.690	0.63	-4.30, 5.56	0.802
Dark olive	6.98	5.94, 8.02	**1.9e-39**	5.13	-2.71, 12.97	0.200	1.50	-3.61, 6.61	0.564
Brown	8.93	6.92, 10.93	**2.9e-18**	8.11	0.59, 15.63	**0.034**	3.23	-1.60, 8.05	0.190
Missing	5.41	4.22, 6.59	**3.3e-19**	6.79	-1.25, 14.84	0.098	1.26	-4.19, 6.72	0.649
Black	**	**		9.47	1.95, 17.00	**0.014**	4.96	-5.03, 14.95	0.330
**Hair colour**									
Blonde	—	—		—	—		—	—	
Red	3.51	2.87, 4.14	**3.0e-27**	7.35	-8.93, 23.63	0.376	**	**	
Light brown	2.12	1.74, 2.50	**5.4e-28**	-11.79	-26.20, 2.62	0.109	10.01	-1.01, 21.03	0.075
Dark brown	4.86	4.46, 5.25	**1.1e-129**	-1.13	-14.48, 12.21	0.868	13.22	2.46, 23.98	**0.016**
Black	6.38	5.72, 7.04	**8.2e-79**	0.43	-12.86, 13.73	0.949	15.26	4.51, 26.01	**0.005**
Other	1.87	0.77, 2.98	**8.9e-04**	3.33	-11.19, 17.86	0.653	16.71	5.03, 28.38	**0.005**
Missing	2.26	-0.44, 4.96	0.100	4.03	-11.23, 19.30	0.604	19.46	6.34, 32.58	**0.004**
**Refractive status**									
Myopia <-6.00D	—	—		—	—		—	—	
Myopia	1.71	1.12, 2.31	**1.5e-08**	-0.50	-2.98, 1.98	0.693	-0.63	-3.24, 1.98	0.636
Emmetropia	2.18	1.58, 2.78	**9.0e-13**	-0.81	-3.26, 1.64	0.517	-0.85	-3.45, 1.74	0.520
Hyperopia	1.54	0.94, 2.14	**4.1e-07**	-1.67	-4.16, 0.82	0.189	-1.63	-4.30, 1.03	0.230
Hyperopia >+6.00D	-1.05	-2.74, 0.65	0.226	**	**		-5.95	-15.02, 3.11	0.198
**Townsend index of deprivation (quintiles)**									
1 (least deprived)	—	—		—	—		—	—	
2	0.16	-0.15, 0.48	0.307	-2.40	-4.73, -0.08	**0.043**	-0.22	-1.84, 1.40	0.790
3	0.12	-0.20, 0.45	0.456	-1.94	-4.08, 0.20	0.075	0.86	-0.70, 2.41	0.279
4	0.12	-0.22, 0.46	0.480	-1.46	-3.49, 0.57	0.157	0.65	-0.89, 2.18	0.409
5 (more deprived)	0.86	0.45, 1.26	**3.6e-05**	-1.98	-3.95, -0.02	**0.047**	0.93	-0.72, 2.57	0.269
Missing	-0.58	-3.56, 2.40	0.702	-3.12	-12.48, 6.25	0.514	5.57	-5.14, 16.29	0.308

[*] White ethnic group n = 40,704; Black ethnic group n = 1,135; and Asian ethnic group n = 1,078.
[**] Levels with no observations per ethnic group.

Supplementary Table 6: Prioritised gene set from the discovery UK Biobank genome-wide association study.

Ensembl ID	Gene symbol
ENSG00000198625	*MDM4*
ENSG00000184144	*CNTN2*
ENSG00000174529	*TMEM81*

ENSG00000117222	*RBBP5*
ENSG00000133059	*DSTYK*
ENSG00000133069	*TMCC2*
ENSG00000163545	*NUAK2*
ENSG00000162873	*KLHDC8A*
ENSG00000066027	*PPP2R5A*
ENSG00000065600	*TMEM206*
ENSG00000117691	*NENF*
ENSG00000143801	*PSEN2*
ENSG00000163050	*ADCK3*
ENSG00000172771	*EFCAB12*
ENSG00000129071	*MBD4*
ENSG00000163913	*IFT122*
ENSG00000163914	*RHO*
ENSG00000178804	*H1FOO*
ENSG00000004399	*PLXND1*
ENSG00000151388	*ADAMTS12*
ENSG00000182631	*RXFP3*
ENSG00000164175	*SLC45A2*
ENSG00000137265	*IRF4*
ENSG00000112685	*EXOC2*
ENSG00000188996	*HUS1B*
ENSG00000028839	*TBPL1*
ENSG00000146411	*SLC2A12*
ENSG00000196367	*TRRAP*
ENSG00000198742	*SMURF1*
ENSG00000185467	*KPNA7*
ENSG00000106245	*BUD31*

ENSG00000106246	*PTCD1*
ENSG00000248919	*ATP5J2-PTCD1*
ENSG00000198556	*ZNF789*
ENSG00000160908	*ZNF394*
ENSG00000196652	*ZKSCAN5*
ENSG00000021461	*CYP3A43*
ENSG00000106261	*ZKSCAN1*
ENSG00000166529	*ZSCAN21*
ENSG00000166526	*ZNF3*
ENSG00000168090	*COPS6*
ENSG00000166508	*MCM7*
ENSG00000221838	*AP4M1*
ENSG00000106290	*TAF6*
ENSG00000197093	*GAL3ST4*
ENSG00000213420	*GPC2*
ENSG00000066923	*STAG3*
ENSG00000160844	*GATS*
ENSG00000214300	*SPDYE3*
ENSG00000121716	*PILRB*
ENSG00000085514	*PILRA*
ENSG00000078487	*ZCWPW1*
ENSG00000146834	*MEPCE*
ENSG00000160813	*PPP1R35*
ENSG00000185955	*C7orf61*
ENSG00000166925	*TSC22D4*
ENSG00000166924	*NYAP1*
ENSG00000077080	*ACTL6B*
ENSG00000172354	*GNB2*

ENSG00000146830	*GIGYF1*
ENSG00000196411	*EPHB4*
ENSG00000146828	*SLC12A9*
ENSG00000087087	*SRRT*
ENSG00000087085	*ACHE*
ENSG00000205277	*MUC12*
ENSG00000169876	*MUC17*
ENSG00000128581	*RABL5*
ENSG00000107165	*TYRP1*
ENSG00000153714	*LURAP1L*
ENSG00000110693	*SOX6*
ENSG00000110075	*PPP6R3*
ENSG00000132749	*MTL5*
ENSG00000197345	*MRPL21*
ENSG00000162341	*TPCN2*
ENSG00000172927	*MYEOV*
ENSG00000123892	*RAB38*
ENSG00000168959	*GRM5*
ENSG00000077498	*TYR*
ENSG00000086991	*NOX4*
ENSG00000172572	*PDE3A*
ENSG00000134532	*SOX5*
ENSG00000080166	*DCT*
ENSG00000152749	*GPR180*
ENSG00000104044	*OCA2*
ENSG00000128731	*HERC2*
ENSG00000183629	*GOLGA8G*
ENSG00000188626	*GOLGA8M*

ENSG00000034053	*APBA2*
ENSG00000184009	*ACTG1*
ENSG00000186765	*FSCN2*
ENSG00000185504	*C17orf70*
ENSG00000182446	*NPLOC4*
ENSG00000185527	*PDE6G*
ENSG00000204237	*OXLD1*
ENSG00000185298	*CCDC137*
ENSG00000214087	*ARL16*
ENSG00000185359	*HGS*
ENSG00000262814	*MRPL12*
ENSG00000262660	*SLC25A10*
ENSG00000183048	*SLC25A10*

Supplementary Table 7: EPIC-Norfolk cohort GWAS results. P values are two-sided and calculated from the chi-squared statistic. The significance threshold for genome-wide significance is p<5E-08.

**Rs identifier**	**chr:pos [hg19]**	**EA/OA (EAF)**	**Beta (95% CI)**	**P**	**Nearest gene**	**Genome-wide significant**
rs6670870	1:205155177	A/T (0.76)	-0.02 (-0.07; 0.03)	4.1E-01	*DSTYK*	
rs173273	1:212446689	G/T (0.41)	–	–	*PPP2R5A*	
rs762948237	3:129178587	TCTTC/T (0.87)	–	–	*IFT122*	

rs16891982	5:33951693	C/G (0.02)	0.59 (0.47; 0.71)	5.1E-22	*SLC45A2*	Yes
rs12203592	6:396321	C/T (0.79)	0.13 (0.07; 0.18)	4.2E-06	*IRF4*	
rs62425803	6:134330249	G/A (0.81)	0.04 (-0.01; 0.09)	1.4E-01	*TCF21*	
rs117756744	7:100277212	G/A (0.98)	0.16 (0.02; 0.29)	2.1E-02	*GNB2*	
rs1325117	9:12613472	G/A (0.36)	0.1 (0.05; 0.14)	5.0E-06	*TYRP1;LURAP1L*	
rs11023814	11:16007053	C/G (0.43)	0.09 (0.04; 0.13)	3.1E-05	*SOX6*	
rs150527451	11:68817897	G/A (0.89)	0.17 (0.11; 0.24)	8.1E-08	*TPCN2*	
rs1060435	11:68855595	A/G (0.59)	0.04 (0; 0.08)	3.5E-02	*TPCN2*	
rs747572	11:87885082	A/G (0.63)	0.05 (0.01; 0.1)	8.6E-03	*CTSC*	
rs1126809	11:89017961	G/A (0.7)	0.06 (0.02; 0.11)	3.7E-03	*TYR*	
rs4762973	12:20710145	A/G (0.75)	0.05 (0; 0.1)	2.8E-02	*PDE3A*	
rs10771034	12:23979199	T/A (0.45)	-0.06 (-0.1; -0.02)	3.3E-03	*SOX5*	
rs766338951	13:95169060	CT/C (0.69)	–	–	*DCT*	
rs1800407	15:28230318	C/T (0.91)	0.14 (0.06; 0.21)	3.4E-04	*OCA2*	

rs12913832	15:28365618	A/G (0.22)	0.53 (0.48; 0.58)	1.2E-98	*HERC2*	Yes
rs7220155	17:79606020	C/T (0.62)	-0.07 (-0.11; -0.02)	1.7E-03	*TSPAN10*	
rs1785433	21:44783282	A/G (0.65)	-0.05 (-0.1; -0.01)	9.0E-03	*SIK1*	

Supplementary Table 8: PheWAS table of results for the UK Biobank cohort.

Table of retinal pigment score phenome-wide association study results meeting the nominal significance threshold (p<0.05) calculated as two-sided p-values calculated with the chi-squared test statistic.

**category**	**phenotype**	**n_cases**	**n_controls**		**pOR (95% CI)**
**Benign Neoplasm/CIN**					
	Benign neoplasm and polyp of uterus	1,042	36,025	0.0230	1.08 (1.01; 1.15)
**Cancers**					
	Primary Malignancy_Other Skin and subcutaneous tissue	2,334	34,733	0.0000	0.9 (0.86; 0.94)
	Primary Malignancy_Malignant Melanoma	644	36,423	0.0172	0.91 (0.83; 0.98)
**Circulatory System**					
	Myocardial infarction	1,482	35,585	0.0165	1.07 (1.01; 1.13)
	Venous thromboembolic disease (Excl PE)	1,095	35,972	0.0232	1.07 (1.01; 1.14)
**Digestive System**					

	Gastro-oesophageal reflux disease	5,622	31,445	0.0015	0.95 (0.93; 0.98)
	Abdominal Hernia	4,355	32,712	0.0027	1.05 (1.02; 1.09)
	Diverticular disease of intestine (acute and chronic)	4,484	32,583	0.0198	1.04 (1.01; 1.07)
**Eye**					
	Diabetic ophthalmic complications	629	36,438	0.0293	0.91 (0.84; 0.99)
	Anterior and Intermediate Uveitis	205	36,862	0.0324	0.86 (0.74; 0.99)
	Retinal detachments and breaks	449	36,618	0.0480	0.91 (0.82; 1)
**Genitourinary system**					
	Postcoital and contact bleeding	323	36,744	0.0315	0.88 (0.79; 0.99)
	Erectile dysfunction	1,201	35,866	0.0363	0.94 (0.88; 1)
**Haematological/Immunological conditions**					
	Agranulocytosis	490	36,577	0.0020	0.86 (0.78; 0.95)
**Infectious Diseases**					
	Infection of skin and subcutaneous tissues	1,462	35,605	0.0004	1.1 (1.04; 1.16)
	Lower Respiratory Tract Infections	3,035	34,032	0.0024	1.06 (1.02; 1.1)
	Bacterial Diseases (excl TB)	5,091	31,976	0.0090	1.04 (1.01; 1.07)

	Other or unspecified infectious organisms	4,922	32,145	0.0219	1.04 (1; 1.07)
**Mental Health Disorders**					
	Schizophrenia, schizotypal and delusional disorders	154	36,913	0.0026	1.26 (1.08; 1.47)
**Musculoskeletal conditions**					
	Fracture of hip	314	36,753	0.0062	1.17 (1.04; 1.31)
	Spondylolisthesis	213	36,854	0.0063	1.2 (1.05; 1.38)
	Carpal tunnel syndrome	1,673	35,394	0.0252	1.06 (1.01; 1.11)
**Neurological conditions**					
	Migraine	2,462	34,605	0.0000	0.91 (0.87; 0.95)
**Respiratory System**					
	COPD	1,664	35,403	0.0001	1.11 (1.05; 1.16)
	Allergic and chronic rhinitis	4,310	32,757	0.0002	0.94 (0.91; 0.97)
	Chronic sinusitis	2,992	34,075	0.0073	0.95 (0.91; 0.99)
	Pulmonary collapse (excl pneumothorax)	450	36,617	0.0417	1.1 (1; 1.21)
**Skin conditions**					
	Actinic keratosis	1,058	36,009	0.0000	0.87 (0.81; 0.93)

Rosacea	857	36,210	0.0019	0.9 (0.83; 0.96)
Seborrheic dermatitis	967	36,100	0.0327	0.93 (0.87; 1)

Supplementary Table 9. Details of summary-level data used for Mendelian randomization analyses.

Trait	Source	Description	Participants (cases / controls)
**Exposure**			
Retinal pigment score	UK Biobank	-	37 067
**Outcome**			
Dermatological			
Actinic keratosis	FinnGen	finngen_R8_L12_ACTINKERA	(9 319 / 331 962)
Basal cell carcinoma of skin	FinnGen	finngen_R8_C3_BASAL_CELL_CARCINOMA_EXALLC	(16 328 / 259 583)
Squamous cell carcinoma of skin	FinnGen	finngen_R8_C3_SQUAMOUS_CELL_CARCINOMA_SKIN_EXALLC	(2 749 / 259 583)
Malignant melanoma of skin	FinnGen	finngen_R8_C3_MELANOMA_SKIN_EXALLC	(2 705 / 259 583)
Other non-melanoma skin cancer	FinnGen	finngen_R8_C3_OTHER_SKIN_EXALLC	(14 863 / 259 583)
Other			
COPD	FinnGen	finngen_R8_J10_COPD	(16 410 / 283 589)

Migraine	FinnGen	finngen_R8_G6_MIGRAINE	(15 905 / 264 662)

Abbreviations: SNP = single nucleotide polymorphism; IV = instrumental variable; COPD = chronic obstructive pulmonary disease.

Supplementary Table 10. Mendelian randomization results for potentially causal associations between retinal pigment score with selected outcomes of interest.

MR method	Actinic keratosis		Basal cell carcinoma of skin		Squamous cell carcinoma of skin		Malignant melanoma of skin		Non-melanoma skin cancer		Chronic obstructive pulmonary disease		Migraine	
	OR (95% CI)	Z statistic (P-value)	OR (95% CI)	Z statistic (P-value)	OR (95% CI)	Z statistic (P-value)	OR (95% CI)	Z statistic (P-value)	OR (95% CI)	Z statistic (P-value)	OR (95% CI)	Z statistic (P-value)	OR (95% CI)	Z sta[illegible] (P-va[illegible]
IVW	0.44 (0.24, 0.83)	-2.55 (**1.06E-02**)	0.59 (0.38, 0.92)	-2.35 (**1.86E-02**)	0.38 (0.20, 0.73)	-2.93 (**3.43E-03**)	0.40 (0.22, 0.73)	-3.00 (**2.69E-03**)	0.60 (0.38, 0.94)	-2.25 (**2.45E-02**)	1.08 (0.95, 1.23)	1.18 (2.39E-01)	1.03 (0.87, 1.23)	0.[illegible] (7.26[illegible]
Weighted median	0.61 (0.47, 0.79)	-3.69 (**2.23E-04**)	0.65 (0.54, 0.78)	-4.75 (**2.01E-06**)	0.54 (0.36, 0.80)	-3.06 (**2.19E-03**)	0.44 (0.31, 0.62)	-4.68 (**2.82E-06**)	0.66 (0.55, 0.80)	-4.21 (**2.56E-05**)	1.11 (0.97, 1.27)	1.46 (1.44E-01)	0.97 (0.83, 1.14)	-0.[illegible] (7.21[illegible]
Weighted mode	0.63 (0.51, 0.78)	-4.15 (**1.35E-03**)	0.69 (0.58, 0.81)	-4.43 (**8.21E-04**)	0.48 (0.32, 0.73)	-3.51 (**4.34E-03**)	0.44 (0.32, 0.61)	-4.86 (**3.94E-04**)	0.71 (0.59, 0.87)	-3.40 (**5.28E-03**)	1.13 (0.98, 1.30)	1.64 (1.27E-01)	0.95 (0.79, 1.14)	-0.[illegible] (5.88[illegible]
MR-Egger	0.28 (0.09, 0.86)	-2.22 (**2.66E-02**)	0.43 (0.20, 0.94)	-2.13 (**3.31E-02**)	0.26 (0.08, 0.85)	-2.24 (**2.52E-02**)	0.45 (0.15, 1.33)	-1.45 (1.48E-01)	0.45 (0.20, 0.99)	-1.98 (**4.78E-02**)	1.20 (0.97, 1.48)	1.66 (9.64E-02)	1.01 (0.74, 1.36)	0.[illegible] (9.71[illegible]

Abbreviations: MR = Mendelian randomization; OR = odds ratio; CI = confidence interval; IVW = inverse-variance weighted.

Odds ratios are expressed per standard deviation increase in retinal pigment score. P-values are two-sided, calculated from the Z-statistic, and values in bold indicate statistically significant results.

Supplementary Table 11. Tests of heterogeneity, directional pleiotropy, and regression dilution statistics for the retinal pigment score instrumental variable.

**MR method**	**Actinic keratosis**		**Basal cell carcinoma of skin**		**Squamous cell carcinoma of skin**		**Malignant melanoma of skin**		**Other non-melanoma skin cancer**		**Chronic obstructive pulmonary disease**		**Migraine**	
	**Estimate**	**P-value**	**Estimate**	**P-value**	**Estimate**	**P-value**	**Estimate**	**P-value**	**Estimate**	**P-value**	**Estimate**	**P-value**	**Estimate**	**P-value**
**IVW**														
Cochran's *Q* statistic	280.3 (12)	6.49E-53	217.5 (12)	7.72E-40	87.4 (12)	1.55E-13	77.7 (12)	1.14E-11	211.7 (12)	1.23E-38	20.3 (12)	6.20E-02	37.7 (12)	1.73E-04
I^2 statistic	95.7%	-	94.5%	-	86.3%	-	84.6%	-	94.3%	-	40.8%	-	68.1%	-
**MR-Egger**														
Rucker's *Q'* statistic	259.1 (11)	3.50E-49	200.7 (11)	5.42E-37	83.1 (11)	3.70E-13	77.3 (11)	4.85E-12	198.3 (11)	1.64E-36	18.0 (11)	8.19E-02	37.5 (11)	9.37E-05

I^2_{GX} statistic	94.9%	-	95.3%	-	94.8%	-	95.5%	-	95.3%	-	96.1%	-	96.2%	-
Intercept	0.05	3.43E-01	0.03	3.36E-01	0.04	4.49E-01	-0.01	8.23E-01	0.03	3.89E-01	-0.01	2.36E-01	0.00	8.39E-01

Abbreviations: MR = Mendelian randomization; IVW = inverse-variance weighted.
Cochran's Q and Rucker's Q' are based on a chi-square distribution. The first value is the test statistic; the value in parentheses indicates the number of degrees of freedom.
The MR-Egger intercept test P-value is calculated from the Z statistic. P-values are two-sided.

Supplementary Figure 1: Percentage of fundus image area covered by algorithm segmentation mask.

A: percentage of vessel and optic disc mask by ethnicity and retinal pigment score (RPS) quintiles; B: percentage of vessel and optic disc mask by ethnicity; C: percentage of vessel mask by ethnicity; D: percentage of optic disc mask by ethnicity.

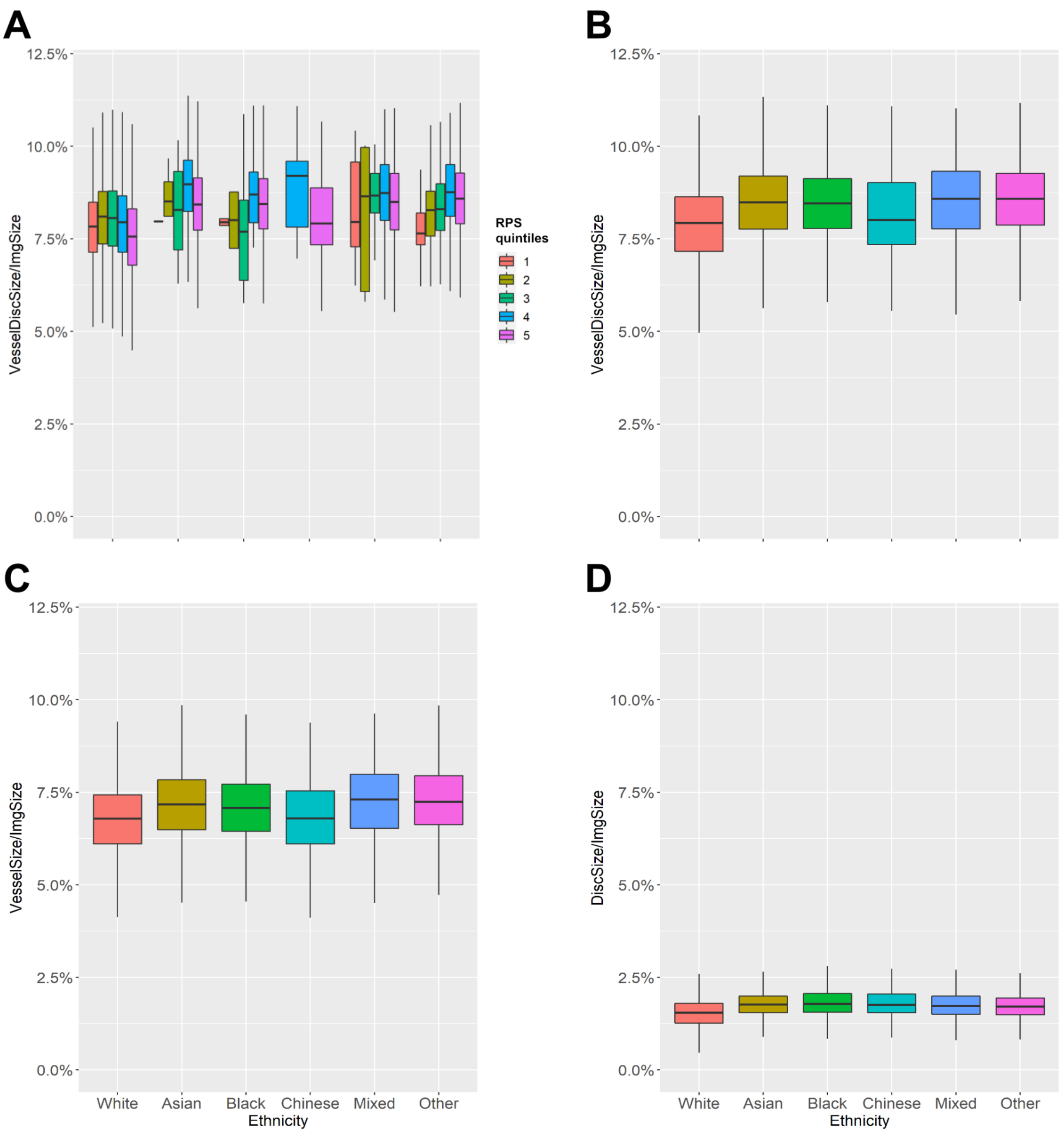

Supplementary Figure 2: Differences in retinal pigment score (RPS) of dilated or eroded vessel and disc masks compared to original masks. There were 100 randomly selected fundus photos from each self-reported ethnicity from the UK Biobank cohort, each of these masks underwent a random erosion or dilation and then the RPS was calculated from these new masks and compared to the RPS calculated from the original mask. Source data are provided as a Source Data file.

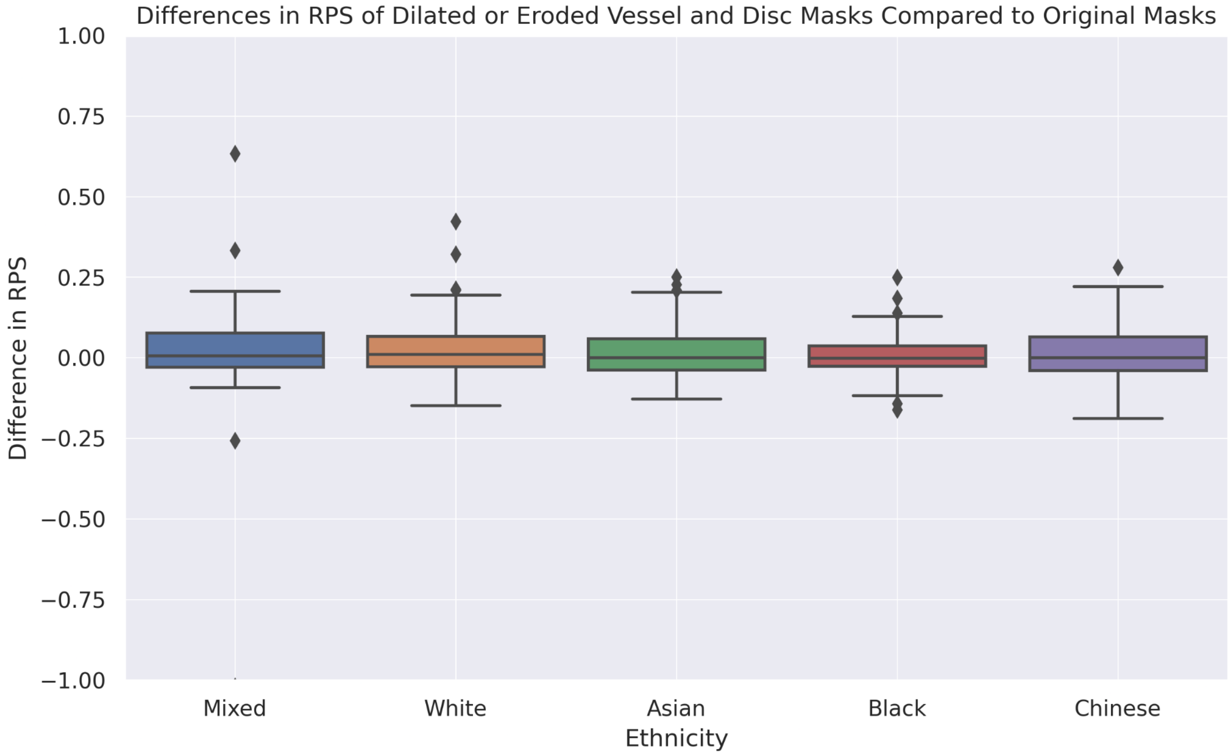

Supplementary Figure 3: Adjusted Regression plots showing the relationship between mean retinal pigment score and covariates of interest. Adjusted means (solid black dots), 95% confidence intervals (vertical solid lines), and regression line for continuous variables (dotted line) are from a linear regression model adjusting for age, sex, and UK Biobank centre. *M, myopia; E, emmetropia; H, hyperopia. Source data are provided as a Source Data file.

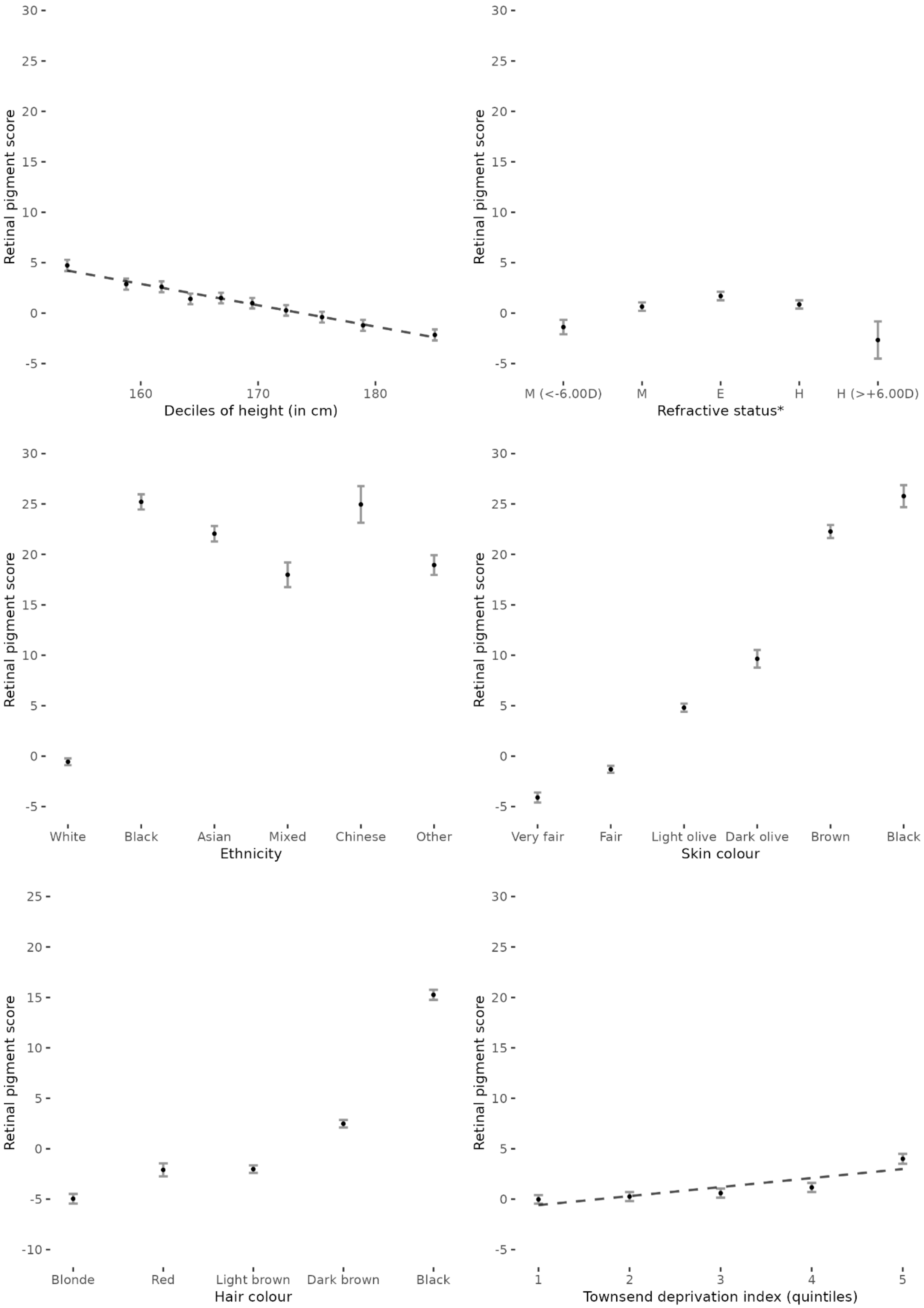
Retinal pigment score
30
25
20
15
10
5
0
-5
160
170
180
Deciles of height (in cm)
M (<-6.00D)
M
E
H
H (>+6.00D)
Refractive status*
White
Black
Asian
Mixed
Chinese
Other
Ethnicity
Very fair
Fair
Light olive
Dark olive
Brown
Black
Skin colour
-10
Blonde
Red
Light brown
Dark brown
Black
Hair colour
1
2
3
4
5
Townsend deprivation index (quintiles)

Supplementary figure 4. Adjusted regression plots for the three main ethnic groups for deciles of age and height.
Adjusted retinal pigment score means (solid black dots), 95% confidence intervals (vertical solid lines), and regression line (dotted line) are from a linear regression model adjusting for sex, and UK biobank centre.

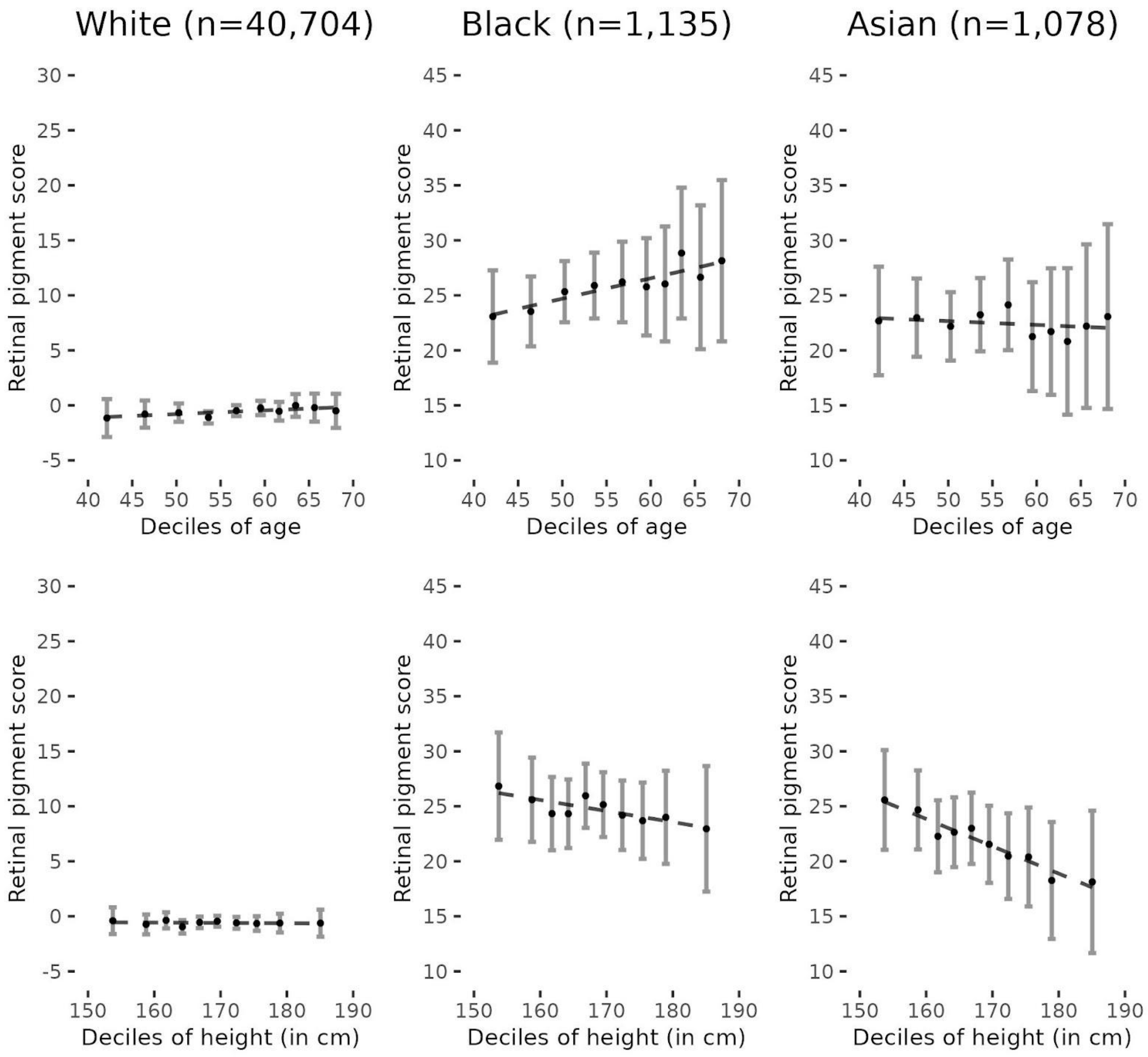

Supplementary Figure 5. Significantly enriched prioritised genes from UK Biobank discovery cohort. Traits that are significantly enriched for prioritised genes from the discovery genome-wide association study (GWAS), as evidenced by previously reported gene-trait associations in the GWAS Catalog. Source data are provided as a Source Data file.

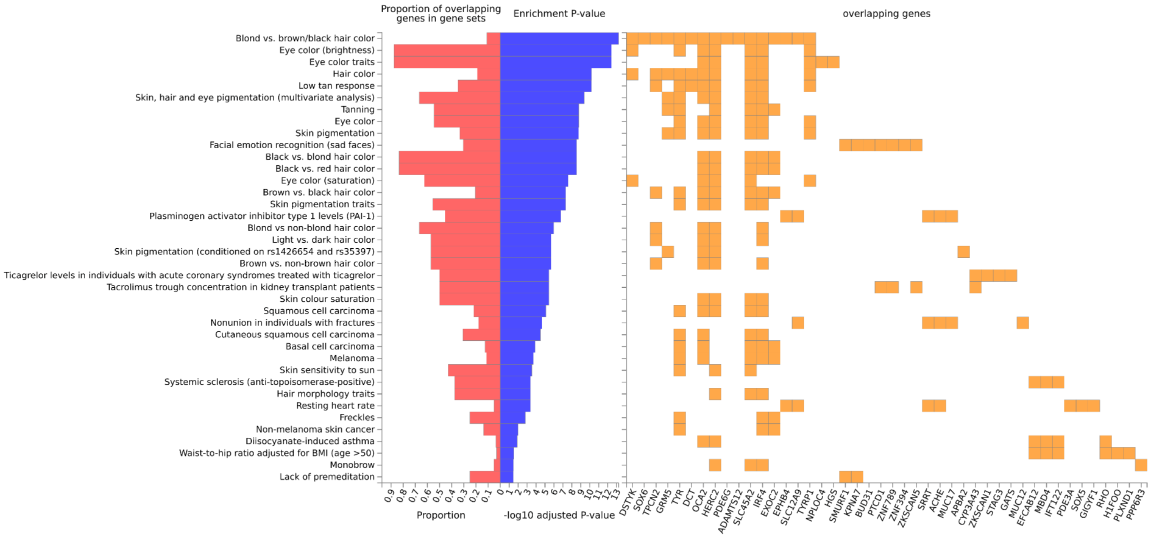

Supplementary Figure 6. Gene Ontology biological pathways from MsigDB significantly enriched for prioritised genes from the discovery genome-wide association study. Source data are provided as a Source Data file.

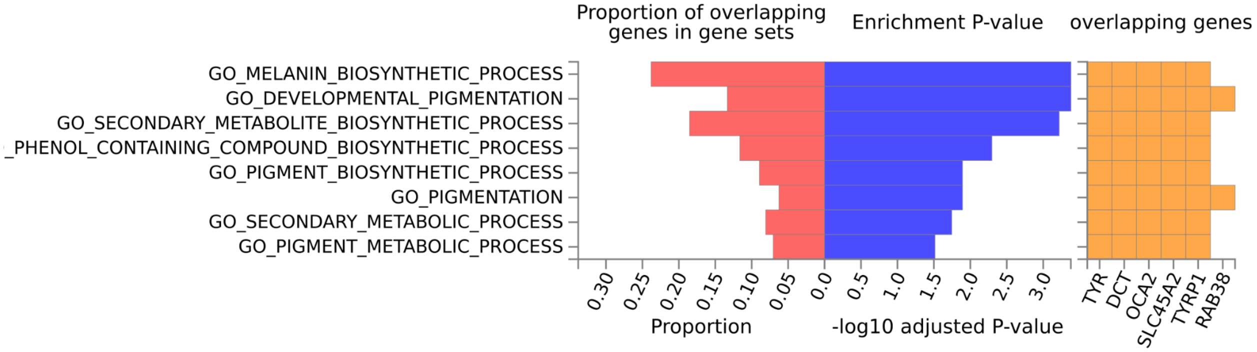

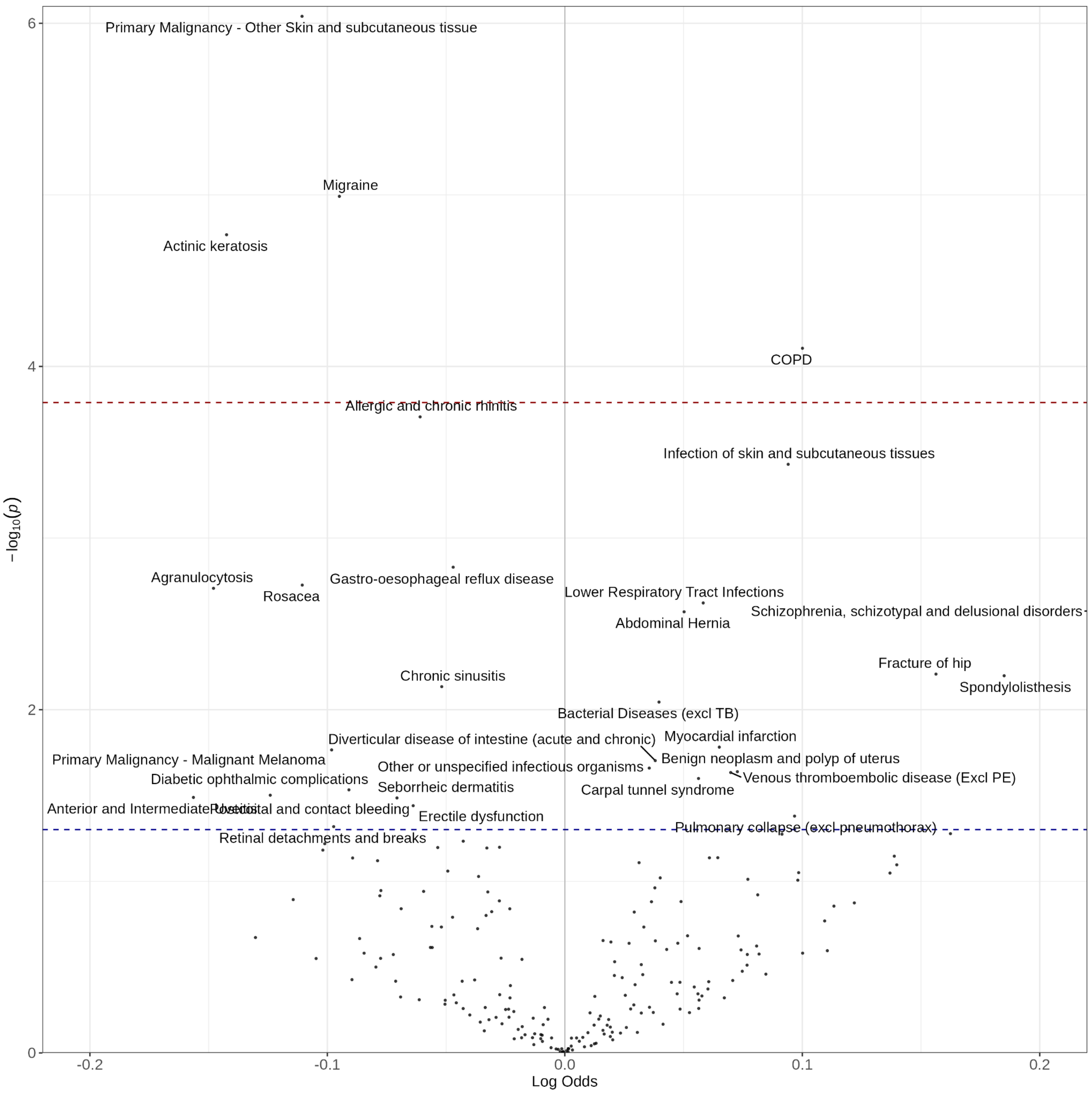

Supplementary Figure 7. Volcano plot summarising phenome-wide association study results, showing potential associations between retinal pigment score (RPS) with 308 health conditions in the UK Biobank cohort. The dashed blue line indicates p=0.05, while the dashed red line indicates the Bonferroni-adjusted significance threshold p=0.05/308 tests. Log odds are per standard deviation increase in RPS. P-values are two sided and calculated with the chi-square test statistic . Abbreviations: TB (tuberculosis); PE (pulmonary embolism); COPD (chronic obstructive pulmonary disease). Source data are provided as a Source Data file.

Supplementary Figure 8. Mendelian randomization results for the association between retinal pigment score (exposure) with actinic keratosis (outcome). Source data are provided as a Source Data file.

Actinic keratosis

a) Individual SNP forest plot

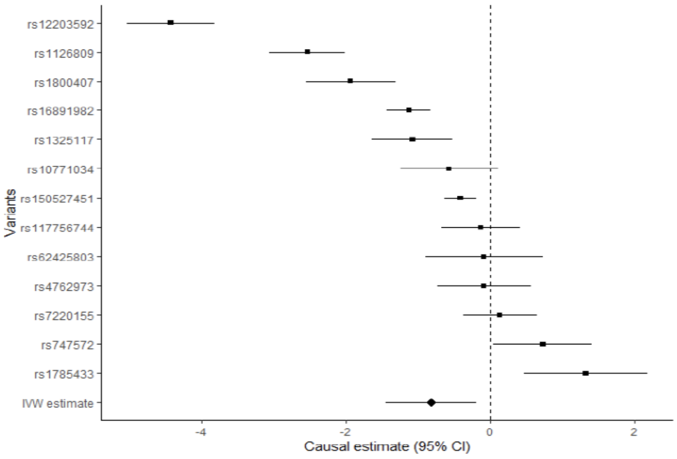

b) Leave-one-out analysis

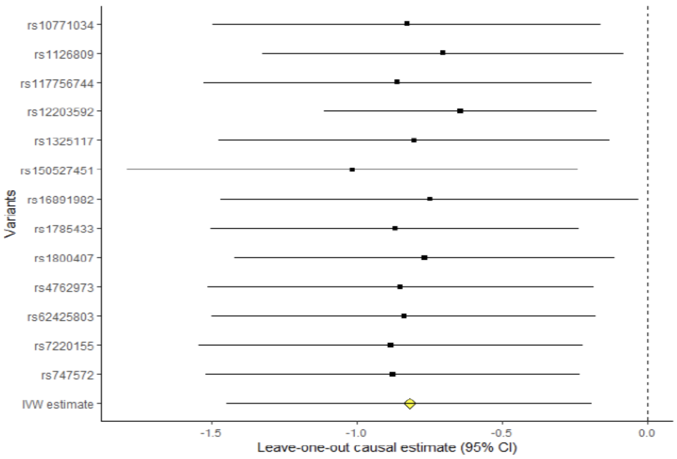

c) MR scatter plot

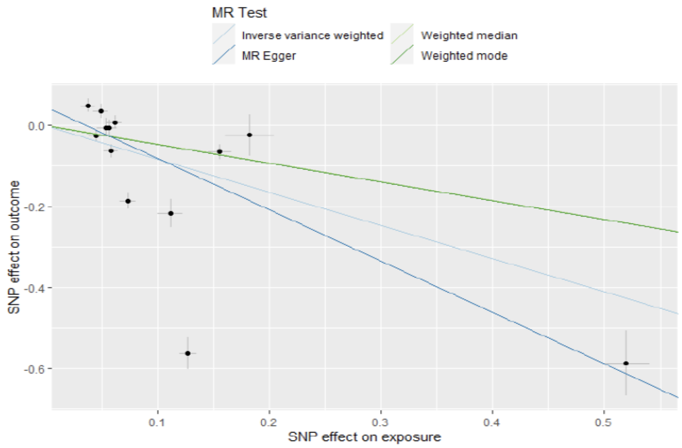

Supplementary Figure 9. Mendelian randomization (MR) results for the association between retinal pigment score (exposure) with basal cell carcinoma (outcome). Abbreviations: SNP (single nucleotide polymorphism). Source data are provided as a Source Data file.

Basal cell carcinoma of skin

a) Individual SNP forest plot

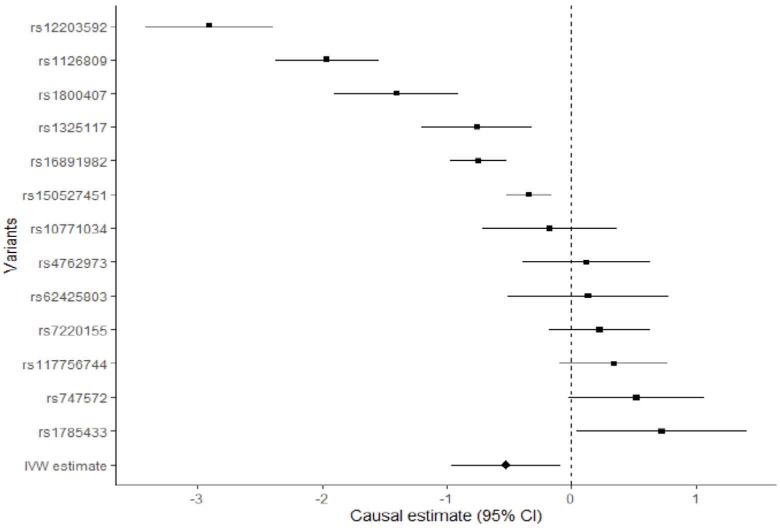

b) Leave-one-out analysis

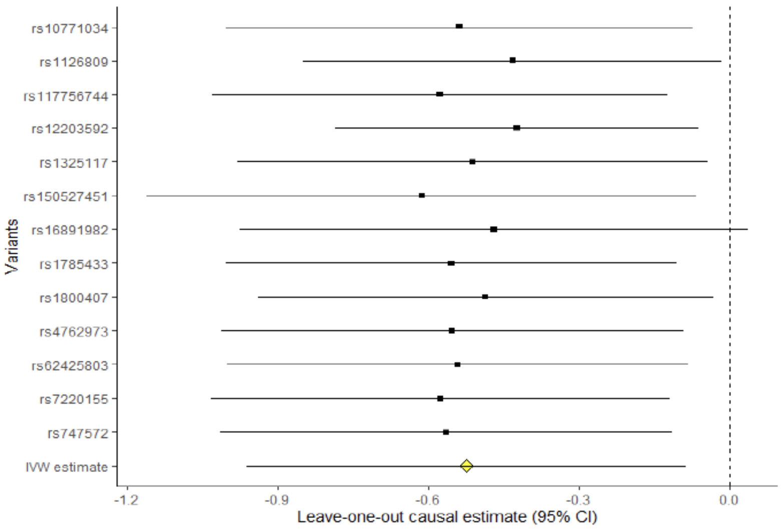

c) MR scatter plot

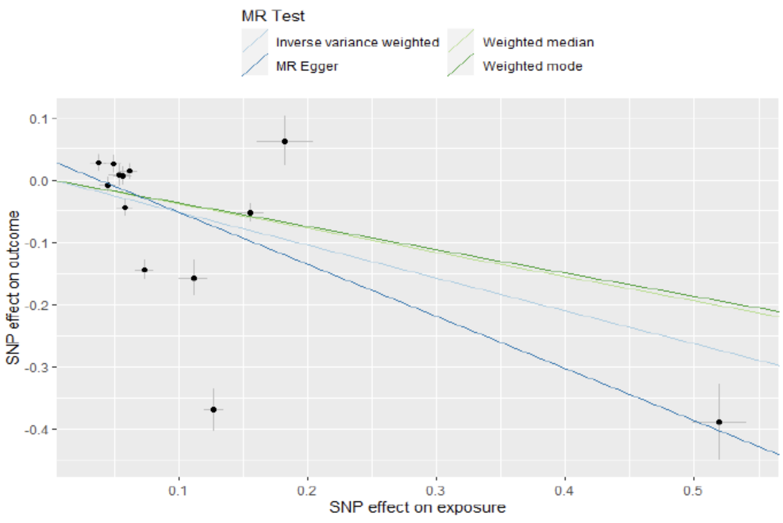

Supplementary Figure 10. Mendelian randomization (MR) results for the association between retinal pigment score (exposure) with squamous cell carcinoma (outcome). Abbreviations: SNP (single nucleotide polymorphism). Source data are provided as a Source Data file.

Squamous cell carcinoma of skin

a) Individual SNP forest plot

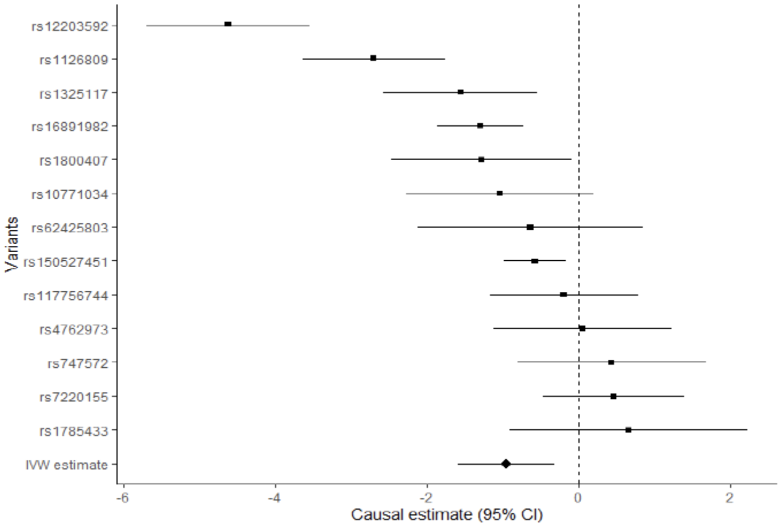

b) Leave-one-out analysis

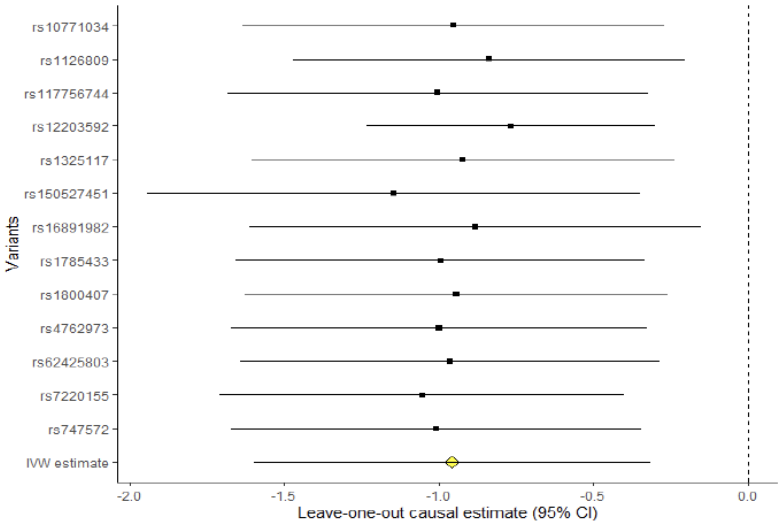

c) MR scatter plot

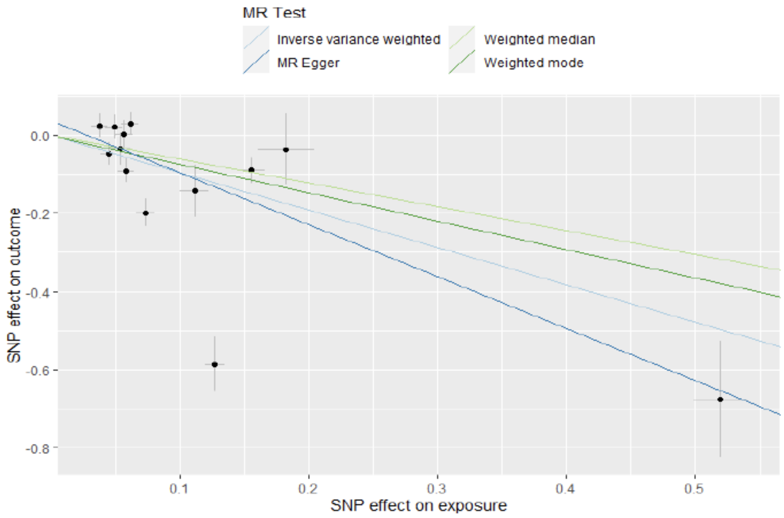

Supplementary Figure 11. Mendelian randomization (MR) results for the association between retinal pigment score (exposure) with malignant melanoma (outcome). Abbreviation: SNP (Single nucleotide polymorphism). Source data are provided as a Source Data file.

Malignant melanoma of skin

a) Individual SNP forest plot

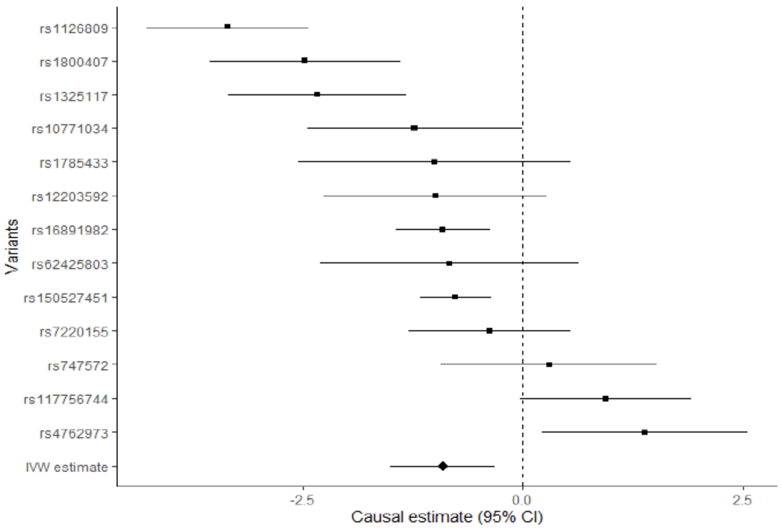

b) Leave-one-out analysis

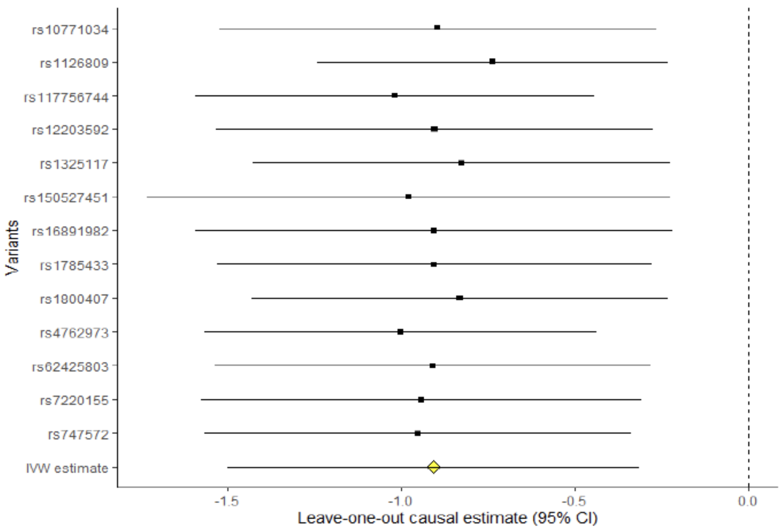

c) MR scatter plot

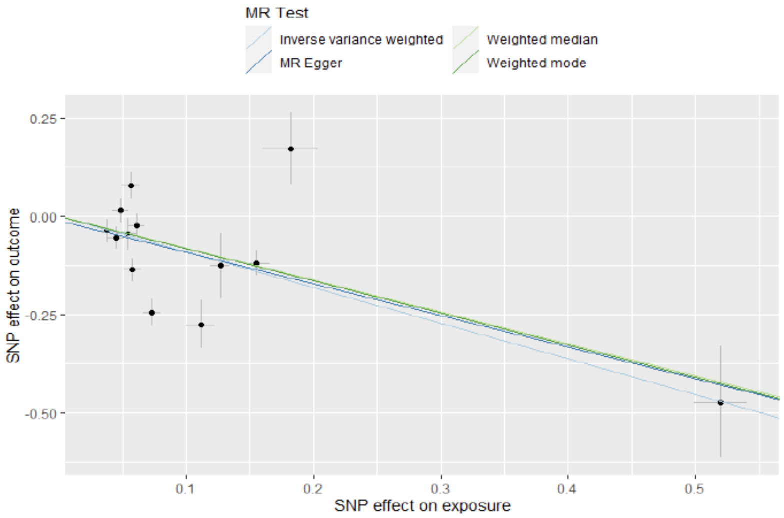

Supplementary Figure 12. Mendelian randomization (MR) results for the association between retinal pigment score (exposure) with other non-melanoma skin cancer (outcome). Abbreviation: SNP (single nucleotide polymorphism). Source data are provided as a Source Data file.

Other non-melanoma skin cancer

a) Individual SNP forest plot

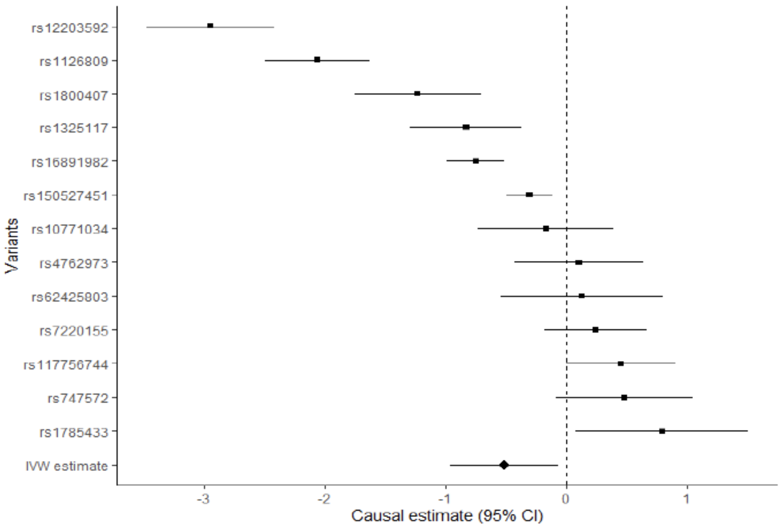

b) Leave-one-out analysis

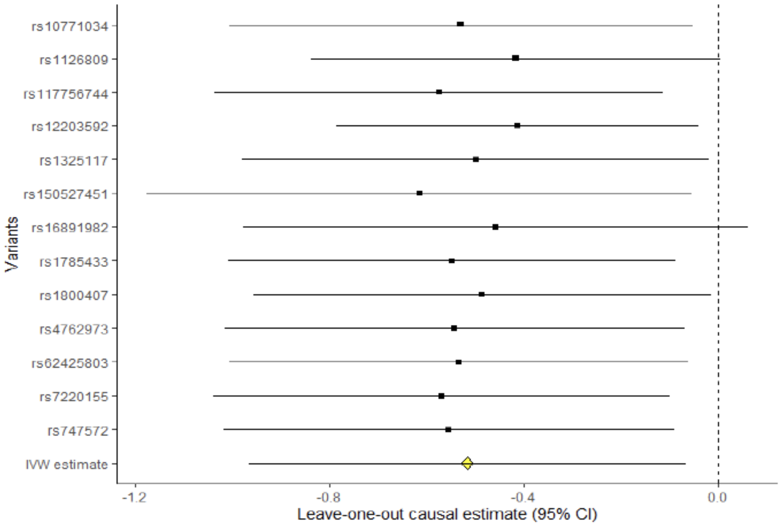

c) MR scatter plot

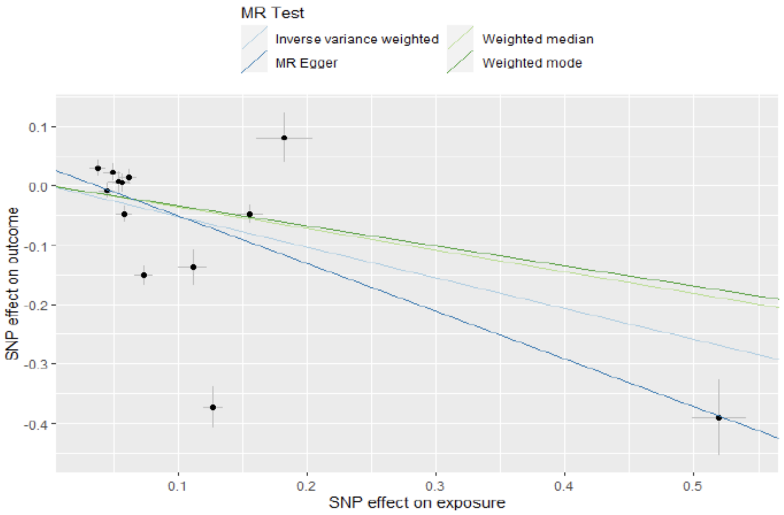

Supplementary Figure 13. Mendelian randomization (MR) results for the association between retinal pigment score (exposure) with chronic obstructive pulmonary disease (outcome). Abbreviation: SNP (single nucleotide polymorphism). Source data are provided as a Source Data file.

Chronic obstructive pulmonary disease

a) Individual SNP forest plot

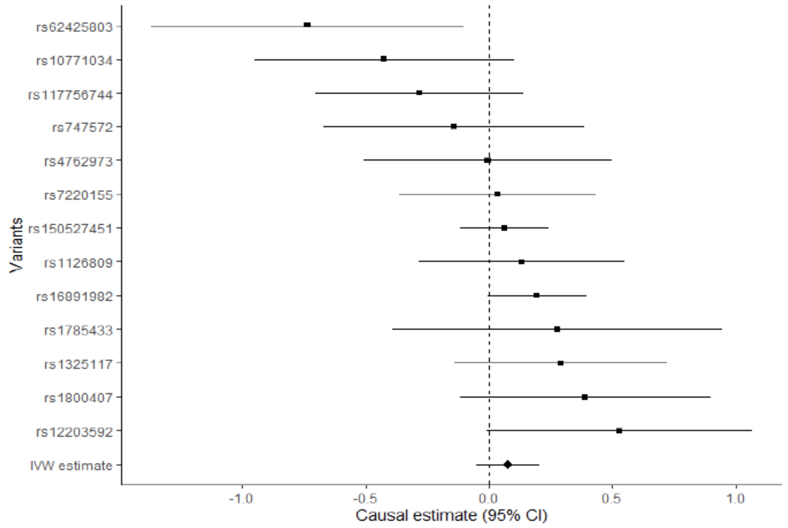

b) Leave-one-out analysis

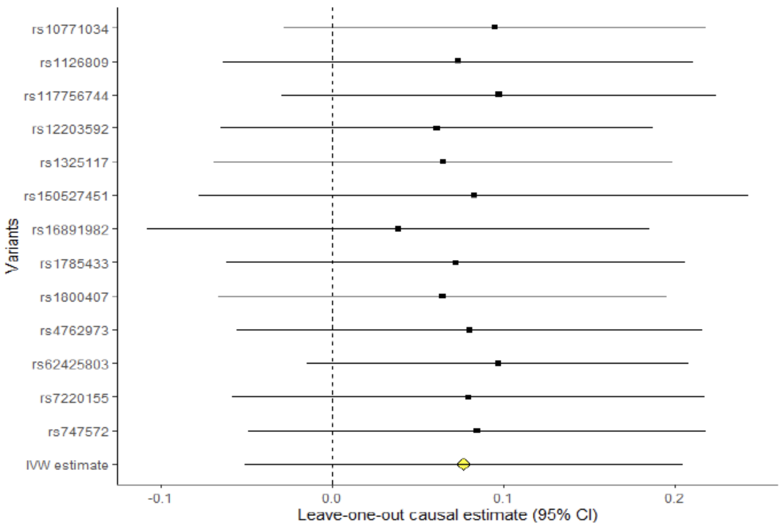

c) MR scatter plot

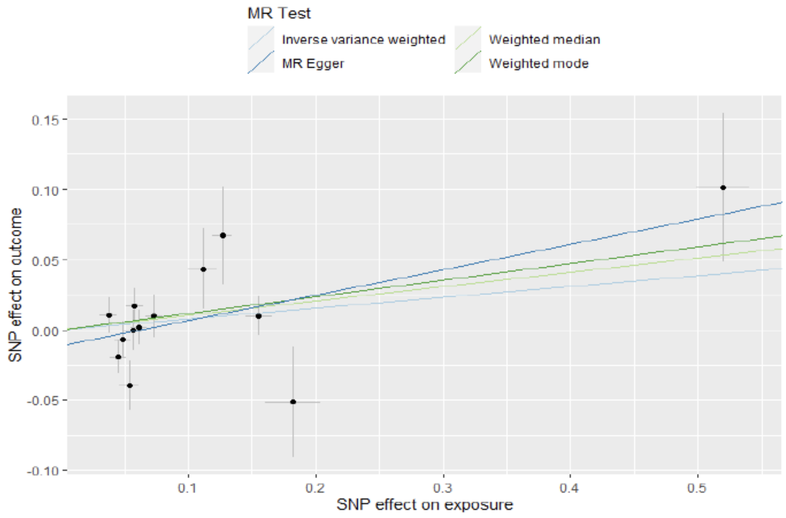

Supplementary Figure 14. Mendelian randomization (MR) results for the association between retinal pigment score (exposure) with migraine (outcome). Abbreviation: SNP (single nucleotide polymorphism).Source data are provided as a Source Data file.

Migraine

a) Individual SNP forest plot

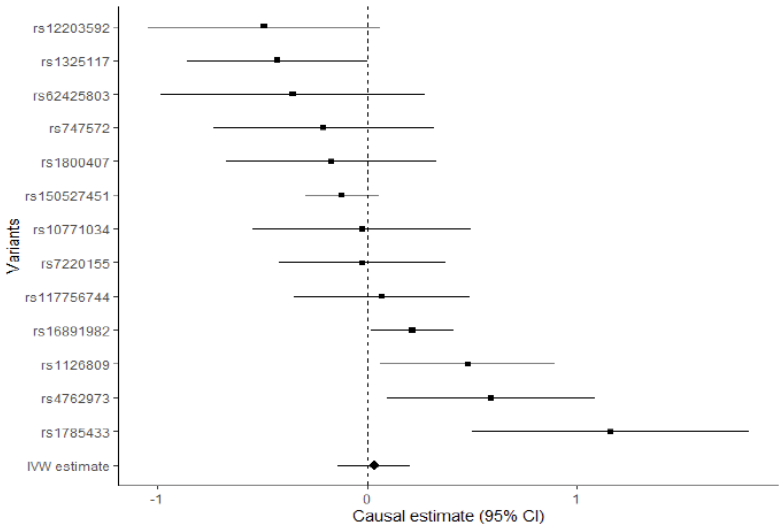

b) Leave-one-out analysis

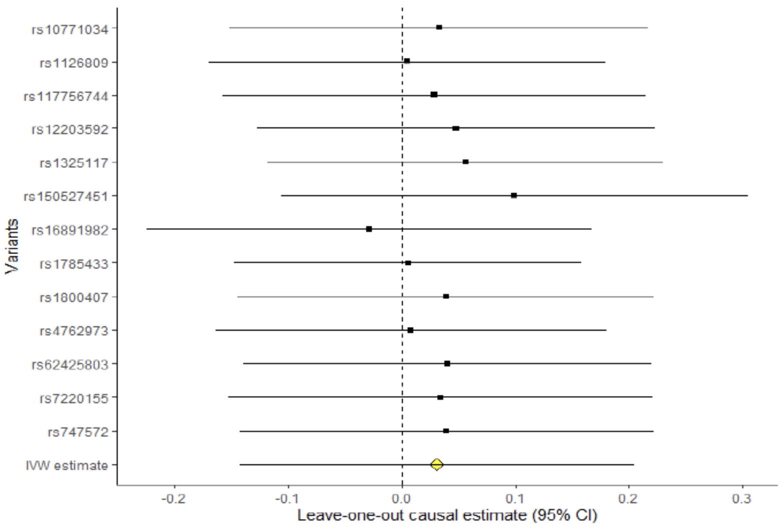

c) MR scatter plot

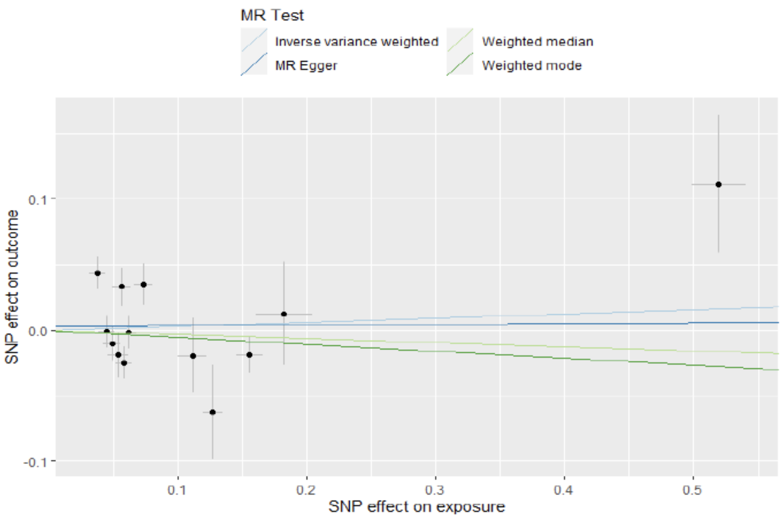

UK Biobank Eye & Vision Consortium

Naomi Allen[11], Tariq Aslam[12], Denize Atan[13], Konstantinos Balaskas[3], Sarah Barman[14], Jenny Barrett[15], Paul Bishop[16], Graeme Black[16], Tasanee Braithwaite[5], Roxana Carare[11], Usha Chakravarthy[17], Michelle Chan[3], Sharon Chua[3], Alexander Day[3], Parul Desai[3], Baljean Dhillon[18], Andrew Dick[13], Alexander Doney[19], Sarah Ennis[20], John Gallacher[21], David (Ted) Garway-Heath[3], Jane Gibson[11], Jeremy Guggenheim[22], Chris Hammond[23], Alison Hardcastle[3], Simon Harding[24], Ruth Hogg[17], Pirro Hysi[23], Gerassimos Lascaratos[25], Thomas Littlejohns[11], Andrew Lotery[26], Phil Luthert[3], Tom MacGillivray[18], Sarah Mackie[15], Savita Madhusudhan[24], Bernadette McGuinness[17], Gareth McKay[17], Martin McKibbin[15], Tony Moore[3], James Morgan[22], Eoin O'Sullivan[25], Richard Oram[27], Chris Owen[28], Praveen Patel[3], Euan Paterson[17], Tunde Peto[17], Axel Petzold[3], Nikolas Pontikos[3], Jugnoo Rahi[29], Alicja Rudnicka[28], Naveed Sattar[30], Jay Self[26], Panagiotis Sergouniotis[16], Sobha Sivaprasad[3], David Steel[31], Irene Stratton[32], Nicholas Strouthidis[3], Cathie Sudlow[33], Zihan Sun[3], Robyn Tapp[34], Dhanes Thomas[3], Emanuele Trucco[19], Ananth Viswanathan[3], Veronique Vitart[33], Mike Weedon[27], Katie Williams[23], Cathy Williams[13], Jayne Woodside[17], Max Yates[35], Yalin Zheng[24].

Affiliations:

1. Department of Ophthalmology, University of Washington, Seattle, WA, USA

2. The Roger and Angie Karalis Johnson Retina Center, Seattle, WA, USA

3. NIHR Biomedical Research Centre, Moorfields Eye Hospital NHS Foundation Trust & University College London Institute of Ophthalmology, London, UK

4. University College London Institute of Cardiovascular Science, London, UK

5. Guy's and St Thomas' NHS Foundation Trust

6. MRC Epidemiology Unit, University of Cambridge, Cambridge, UK

7. International Centre for Eye Health, Faculty of Infectious and Tropical Diseases, London School of Hygiene & Tropical Medicine, London, UK

8. Eye Department, Kilimanjaro Christian Medical Centre, Moshi, United Republic of Tanzania

9. NIHR Birmingham Biomedical Research Centre, Birmingham, UK

10. Lions Eye Institute, University of Western Australia, Nedlands, WA, Australia

11. Nuffield Department of Population Health, University of Oxford, Oxford, UK

12. Manchester Royal Eye Hospital, The University of Manchester, Manchester, UK

13. Bristol Eye Hospital, University of Bristol, Bristol, UK

14. Department of Computer Science and Mathematics, Kingston University, London, UK

15. School of Medicine, University of Leeds, Leeds, UK

16. Division of Evolution, Infection and Genomics, The University of Manchester, Manchester, UK

17. Centre for Public Health, School of Medicine, Dentistry and Biomedical Sciences, Queen's University Belfast, Belfast, UK

18. Centre for Clinical Brain Sciences, University of Edinburgh, Edinburgh, Scotland

19. Pat Macpherson Centre for Pharmacogenomics and Pharmacogenetics, Division of Population Health & Genomics, School of Medicine, University of Dundee, Dundee, UK

20. Human Development and Health, Faculty of Medicine, University Hospital Southampton, Southampton, Hampshire, UK

21. Dementias Platform UK, Oxford, UK

22. School of Optometry & Vision Sciences, Cardiff University, Cardiff, UK

23. Department of Twin Research and Genetic Epidemiology, King's College London, London, UK

24. Department of Eye and Vision Science, Institute of Life Course and Medical Sciences, University of Liverpool, Liverpool, UK

25. Department of Ophthalmology, King's College Hospital NHS Foundation Trust, London, UK

26. Clinical and Experimental Sciences, Faculty of Medicine, University of Southampton, Southampton, UK

27. University of Exeter College of Medicine & Health, Exeter, UK

28. Population Health Research Institute, St George's, University of London, London, UK

29. Population, Policy and Practice Research and Teaching Department, Great Ormond Street Institute of Child Health, University College London, London, UK

30. School of Cardiovascular and Metabolic Health, University of Glasgow, Glasgow, UK

31. Biosciences Institute, Faculty of Medical Sciences, Newcastle University, Newcastle upon Tyne, UK

32. Gloucestershire Retinal Research Group, Cheltenham General Hospital, Gloucestershire Hospitals NHS Foundation Trust, Cheltenham, UK

33. Centre for Medical Informatics, Usher Institute of Population Health Sciences and Informatics, University of Edinburgh, UK

34. Research Centre for Intelligent Health Care, Coventry University, Coventry, UK

35. Norwich Epidemiology Centre, Norwich Medical School, University of East Anglia, Norwich, UK.
