# Supplementary material for: Machine learning derived retinal pigment score from ophthalmic imaging shows ethnicity is not biology
Source: Nat Commun. 2025 Jan 2;16:60. doi: 10.1038/s41467-024-55198-7 (PMC11696055; doi:10.1038/s41467-024-55198-7)
Supplement: Supplementary file 2 — Reporting Summary [file 41467_2024_55198_MOESM2_ESM.pdf]

nature portfolio

Corresponding author(s): Catherine Egan

Last updated by author(s): Aug 17,2024

Reporting Summary

Nature Portfolio wishes to improve the reproducibility of the work that we publish. This form provides structure for consistency and transparency in reporting. For further information on Nature Portfolio policies, see our Editorial Policies and the Editorial Policy Checklist.

Statistics

For all statistical analyses, confirm that the following items are present in the figure legend, table legend, main text, or Methods section.

n/a	Confirmed	
☐	☒	The exact sample size (*n*) for each experimental group/condition, given as a discrete number and unit of measurement
☐	☒	A statement on whether measurements were taken from distinct samples or whether the same sample was measured repeatedly
☐	☒	The statistical test(s) used AND whether they are one- or two-sided<br>*Only common tests should be described solely by name; describe more complex techniques in the Methods section.*
☐	☒	A description of all covariates tested
☐	☒	A description of any assumptions or corrections, such as tests of normality and adjustment for multiple comparisons
☐	☒	A full description of the statistical parameters including central tendency (e.g. means) or other basic estimates (e.g. regression coefficient) AND variation (e.g. standard deviation) or associated estimates of uncertainty (e.g. confidence intervals)
☐	☒	For null hypothesis testing, the test statistic (e.g. *F*, *t*, *r*) with confidence intervals, effect sizes, degrees of freedom and *P* value noted<br>*Give P values as exact values whenever suitable.*
☐	☒	For Bayesian analysis, information on the choice of priors and Markov chain Monte Carlo settings
☐	☒	For hierarchical and complex designs, identification of the appropriate level for tests and full reporting of outcomes
☐	☒	Estimates of effect sizes (e.g. Cohen's *d*, Pearson's *r*), indicating how they were calculated

Our web collection on statistics for biologists contains articles on many of the points above.

Software and code

Policy information about availability of computer code

Data collection	The code used for data collection is available here: https://github.com/uw-biomedical-ml/retinal-pigmentation-score
Data analysis	The code used for data analysis included that which is available here: https://github.com/uw-biomedical-ml/retinal-pigmentation-score. This code includes a link to a docker container with all software versions required to execute the code. Here are the required python packages to execute the code:<br>absl-py==0.13.0<br>art==5.2<br>cached-property==1.5.2<br>cachetools==4.2.2<br>certifi==2021.5.30<br>charset-normalizer==2.0.4<br>cycler==0.10.0<br>#dataclasses==0.8<br>decorator==4.4.2<br>efficientnet-pytorch==0.7.1<br>future==0.18.2<br>google-auth==1.35.0<br>google-auth-oauthlib==0.4.5<br>grpcio==1.39.0<br>h5py==3.1.0

idna==3.2
imageio==2.9.0
importlib-metadata==4.6.4
joblib==1.0.1
kiwisolver==1.3.1
Markdown==3.3.4
matplotlib==3.3.4
networkx==2.5.1
numpy==1.19.5
oauthlib==3.1.1
olefile==0.46
opencv-python==4.5.3.56
pandas==1.1.5
Pillow==8.3.1
protobuf==3.17.3
pyasn1==0.4.8
pyasn1-modules==0.2.8
pycm==3.2
pydicom==2.3.0
pyparsing==2.4.7
python-dateutil==2.8.2
pytz==2021.1
PyWavelets==1.1.1
requests==2.26.0
requests-oauthlib==1.3.0
rsa==4.7.2
scikit-image==0.17.2
scikit-learn==0.24.2
scipy==1.5.4
six==1.16.0
tensorboard==2.6.0
tensorboard-data-server==0.6.1
tensorboard-plugin-wit==1.8.0
threadpoolctl==2.2.0
tifffile==2020.9.3
torch==1.7.0
torchaudio==0.7.0
torchvision==0.8.0
tqdm==4.62.1
typing-extensions==3.10.0.0
urllib3==1.26.6
Werkzeug==2.0.1
zipp==3.5.0

The R and python versions used for analysis are listed in the manuscript. The packages used are also listed in the manuscript.

The GWAS analysis was performed using REGENIE software.83 GCTA-COJO was used to prioritise lead variants.84 Genomic inflation factor and heritability estimates were calculated using the LDSC tool85 and pre-calculated LD scores for European ancestry (https://data.broadinstitute.org/alkesgroup/LDSCORE/eur_w_ld_chr.tar.bz2). All other statistical analyses and were performed in R (R for GNU macOS, Version 4.2.0, The R Foundation for Statistical Computing, Vienna, Austria).86 R packages used included PheWAS87, TwoSampleMR88, MendelianRandomization89, ukbwranglr90, codemapper91, targets92, tarchetypes93, tidyverse 94, workflowr95, flextable96, gtsummary97 and knitr.98

For manuscripts utilizing custom algorithms or software that are central to the research but not yet described in published literature, software must be made available to editors and reviewers. We strongly encourage code deposition in a community repository (e.g. GitHub). See the Nature Portfolio guidelines for submitting code & software for further information.

Data

Policy information about availability of data

All manuscripts must include a data availability statement. This statement should provide the following information, where applicable:

- Accession codes, unique identifiers, or web links for publicly available datasets
- A description of any restrictions on data availability
- For clinical datasets or third party data, please ensure that the statement adheres to our policy

Access to the UK Biobank is restricted to safeguard the privacy of the participants and requires an application. The restrictions depend on the level of access granted. One can apply for access on their website. You cannot share the UK Biobank data with researchers who are not registered with the UK Biobank. Registrations are reviewed within 10 working days of submission. The length of access depends on the access granted.
Access to the EPIC-Norfolk Eye study is restricted and requires an application because of the desire to safeguard the privacy of participants. One can request access via the EPIC-Norfolk Management Committee. The data is available to researchers with relevant scientific and ethics approvals for their research, including those in

other countries and in commercial companies who are looking for new treatments or laboratory tests. Applications are generally reviewed within 1 month.
The Tanzanian fundus photo dataset was transferred to LSHTM under a formal data transfer agreement with the National Institute for Medical Research in Tanzania. This agreement stipulates that the dataset be used for teaching or academic research purposes only. Requests for access to the dataset can be made to Charles Cleland (charles.cleland@lshtm.ac.uk) and replies will be within ten working days. If access to the dataset is granted it will be for a period of six weeks.
The Australian dataset is available under restricted access in order to safeguard participant privacy. This dataset, also known as the Derbarl Yerrigan Health Service data, is a First Nations of Western Australia diabetic screening dataset. This dataset is subject to ethical approval for use by the Ethics Committee operated by the Aboriginal Health Council of Western Australia (AHCWA). A written request will be considered and responses should be returned in less than one month. Access to the data is subject to further ethics applications to AHCWA and the duration of access will depend on the applications. Please reach out to angus.turner@uwa.edu.au for inquiries
The Chinese dataset is a subset of the publically available ODIR dataset and can be accessed as described in the corresponding manuscript.(Li et al. 2021) The link to download the data is: https://odir2019.grand-challenge.org/dataset/
The raw data used in this study are protected and are not available due to data privacy laws. The data generated in this study are provided in the Source Data file. Retinal pigment scores for UK Biobank participants will be made available to approved UK Biobank researchers as a returned dataset (https://biobank.ndph.ox.ac.uk/ukb/docs.cgi?id=1). FinnGen genome-wide association study (GWAS) summary statistics are publicly available online (https://www.finngen.fi/en/access_results). Summary statistics from the GWAS analyses presented in this study will be made publicly available from the NHGRI-EBI Catalog of human genome-wide association studies (https://www.ebi.ac.uk/gwas/).

Research involving human participants, their data, or biological material

Policy information about studies with human participants or human data. See also policy information about sex, gender (identity/presentation), and sexual orientation and race, ethnicity and racism.

Reporting on sex and gender	Sex was included in the UK Biobank dataset. As per the study protocol, the sex varable is a mix of self-reported and assigned sex. Gender information was not used in this manuscript.
Reporting on race, ethnicity, or other socially relevant groupings	Ethnicity was self-reported unless otherwise specified. Ethnicity in the UK-biobank was collected by participants selecting their demographic information from a touchscreen. Ethnicity was not collected in the Chinese, Tanzanian or Australian dataset.
Population characteristics	In the UK Biobank the median age was 56 years old (IQR: 49 - 63). The cohort was 55% female and 45% male. The cohort was 92% White, 2.6% Black, 2.4% Asian, 0.8 % Mixed, 0.4% Chinese, 1.4% Other. Other characteristics can be seen in Supplementary Table 2.
Recruitment	All participants completed a detailed touchscreen questionnaire on demographic, clinical and lifestyle-related information. The choices for ethnic background were categorised as white, mixed, Asian or Asian British, black or black British, Chinese, or other ethnic group. Participant postcode at the time of recruitment was used to determine Townsend Deprivation Index (TDI), based on the corresponding output area from the preceding national census; a higher positive score implies a greater degree of deprivation. Medical history was obtained through verbal interview with a trained nurse, including the date of first diagnosis for non-cancer and cancer illnesses, as well as any major operations. Participants gave broad consent for prospective data linkage to national electronic health records (EHR) and registries, including hospital episode statistics, death register and cancer register. Linkage to primary care records is currently available for approximately 45% of the cohort (~230,000 participants, up to 2016 or 2017 depending on data supplier). Further details of the overall study protocol and protocols for individual tests are available online (https://biobank.ndph.ox.ac.uk/ukb/index.cgi).<br>The EPIC-Norfolk Eye Study is a study of 8623 participants from Norfolk, England and was added onto the EPIC Cohort.29 The EPIC study is a collaborative study involving 10 countries that began participant recruitment in 1989.59 The EPIC-Norfolk, a United Kingdom branch of this study, comprises a population-based cohort of 25 639 participants between 40 and 79 years of age at enrolment recruited from 35 participating general practices in Norfolk, United Kingdom. Baseline examinations were carried out between 1993 and 1997.60<br>The Tanzanian dataset consisted of images acquired from people attending a diabetic eye screening service in the Kilimanjaro region of northern Tanzania between June 2017 and August 2018. All participants were African and had a diagnosis of diabetes mellitus. A total of 2076 retinal photographs of 1345 eyes from 690 people with diabetes comprise the dataset. Only images that were graded as having no, or mild, retinopathy were included in the analyses.<br>The Australian dataset consisted of images acquired from a single Aboriginal Community Controlled Health Service located within a metropolitan area of Perth, Western Australia. Participants were Aboriginal people with diabetes mellitus attending a retinal screening service. Retinal photographs of 1682 eyes of 864 people were acquired consecutively between July 2013 and October 2020. Only images that were graded as having no retinopathy or mild retinopathy were included in this study.<br>The Chinese dataset is the publicly available ODIR dataset. In brief, it is a labelled collection of manually curated colour fundus photos with a wide range of disease and pathology. There are 10,000 images from 5,000 individuals from 487 clinical hospitals in 26 provinces across China. From this dataset, we included only the 3098 normal fundus images.
Ethics oversight	We analysed data from UK Biobank participants who as part of their examinations underwent enhanced ophthalmic review. Ethics approval was obtained by the Northwest Multi-centre Research Ethics Committee (REC reference number 06/MRE08/65; approved project number 28541), our research adhered to the tenets of the Declaration of Helsinki. Informed consent was obtained from all study participants and all participants were free to withdraw from the study at any time.58<br>The EPIC-Norfolk Eye Study was carried out following the principles of the Declaration of Helsinki and the Research Governance Framework for Health and Social Care and was approved by the Norfolk Local Research Ethics Committee (identifier: 05/Q0101/191) and the East Norfolk and Waveney National Health Service Research Governance Committee (identifier: 2005EC07L). All participants gave written informed consent. The study protocol is available online at https://www.epic-norfolk.org.uk/.<br>The Tanzanian imaging data was collected following review and approval by the Tanzanian National Institute for Medical

Research (Reference id: NIMR/HQ/R.8a/Vol.IX/2402), the Kilimanjaro Christian Medical Centre (Reference number: 776), and the London School of Hygiene & Tropical Medicine Ethics Committees (Reference number: 10172).
The Australian dataset was approved by the Western Australian Aboriginal Health Ethics Committee (Reference number: 864).
The Chinese dataset was collected as part of a private dataset and ethical collection was enforced by the original dataset creators. The authors state the publishing of the dataset follows the ethical and privacy rules of China.

Note that full information on the approval of the study protocol must also be provided in the manuscript.

Field-specific reporting

Please select the one below that is the best fit for your research. If you are not sure, read the appropriate sections before making your selection.

- [x] Life sciences
- [ ] Behavioural & social sciences
- [ ] Ecological, evolutionary & environmental sciences

For a reference copy of the document with all sections, see nature.com/documents/nr-reporting-summary-flat.pdf

Life sciences study design

All studies must disclose on these points even when the disclosure is negative.

Sample size	We included all available data and did not use any sampling.
Data exclusions	There were no exclusions applied when creating the retinal pigment scores from the fundus photos.<br><br>In the GWAS the following exclusions were applied for sample quality control: individuals with relatedness corresponding to third-degree relatives or closer, excess of missing genotypes or more heterozygosity than expected. The GWAS analysis was furthermore restricted to individuals of European ethnicity only. The following exclusions were applied for variant-level quality control: call rate <95%, Hardy-Weinberg equilibrium p<1x10-6, posterior call probability <0.9, INFO score <0.9 and minor allele frequency <0.01.
Replication	Replication of the GWAS on the UK Biobank cohort was performed on the EPIC-Norfolk Cohort. Lead variants reaching genome-wide significance (p<5x10-8) in the UK Biobank cohort were re-evaluated in a replication GWAS analysis, conducted in the EPIC-Norfolk cohort. A Bonferroni-adjusted replication significance threshold was set at p=0.05/17.
Randomization	Randomization was not performed in this study.<br><br>Cohorts were allocated into experimental groups based off of self-reported ethnicity for patients in the UK Biobank. The groups of White, Black and Asian were used for additional analysis due to the small sample size Mixed and Chinese participants. The associations of retinal pigment score and various clinical variables were tested by adjusting for the following covariates: age, height, sex, skin colour, hair colour, refractive status, townsend index of deprivation. Variance inflation factor testing was performed to assess for strong collinearity between these covariates.<br><br>Mendelian randomization was performed to evaluate causality between exposure and outcome variables
Blinding	Blinding was not performed in this study.

Reporting for specific materials, systems and methods

We require information from authors about some types of materials, experimental systems and methods used in many studies. Here, indicate whether each material, system or method listed is relevant to your study. If you are not sure if a list item applies to your research, read the appropriate section before selecting a response.

Materials & experimental systems

n/a	Involved in the study	
[x]	[ ]	Antibodies
[x]	[ ]	Eukaryotic cell lines
[x]	[ ]	Palaeontology and archaeology
[x]	[ ]	Animals and other organisms
[x]	[ ]	Clinical data
[x]	[ ]	Dual use research of concern
[x]	[ ]	Plants

Methods

n/a	Involved in the study	
[x]	[ ]	ChIP-seq
[x]	[ ]	Flow cytometry
[x]	[ ]	MRI-based neuroimaging

Plants

Seed stocks	*Report on the source of all seed stocks or other plant material used. If applicable, state the seed stock centre and catalogue number. If plant specimens were collected from the field, describe the collection location, date and sampling procedures.*
Novel plant genotypes	*Describe the methods by which all novel plant genotypes were produced. This includes those generated by transgenic approaches, gene editing, chemical/radiation-based mutagenesis and hybridization. For transgenic lines, describe the transformation method, the number of independent lines analyzed and the generation upon which experiments were performed. For gene-edited lines, describe the editor used, the endogenous sequence targeted for editing, the targeting guide RNA sequence (if applicable) and how the editor was applied.*
Authentication	*Describe any authentication procedures for each seed stock used or novel genotype generated. Describe any experiments used to assess the effect of a mutation and, where applicable, how potential secondary effects (e.g. second site T-DNA insertions, mosiacism, off-target gene editing) were examined.*
